# The Role of Relatedness in the Motivation and Vitality of University Students in Online Classes During Social Distancing

**DOI:** 10.3389/fpsyg.2021.702323

**Published:** 2022-01-25

**Authors:** Vanda Capon-Sieber, Carmen Köhler, Ayşenur Alp Christ, Jana Helbling, Anna-Katharina Praetorius

**Affiliations:** ^1^Faculty of Arts and Social Sciences, Institute of Education, University of Zurich, Zurich, Switzerland; ^2^Leibniz Institute for Research and Information in Education (DIPF), Frankfurt, Germany

**Keywords:** relatedness need satisfaction, relatedness support, affiliation motive, online teaching, COVID-19, motivation, communication channel, vitality

## Abstract

As part of the social distancing measures for preventing the spread of COVID-19, many university courses were moved online. There is an assumption that online teaching limits opportunities for fostering interpersonal relationships and students’ satisfaction of the basic need for relatedness – reflected by experiencing meaningful interpersonal connections and belonging – which are considered important prerequisites for student motivation and vitality. In educational settings, an important factor affecting students’ relatedness satisfaction is the teachers’ behavior. Although research suggests that relatedness satisfaction may be impaired in online education settings, to date no study has assessed how university lecturers’ relatedness support might be associated with student relatedness satisfaction and therefore, student motivation and vitality. This study tested this mediating relationship using data collected during the early days of the COVID-19 pandemic. The study also investigated whether the relations were moderated by a high affiliation motive which reflects a dispositional wish for positive and warm relationships. The possible importance of the communication channel selected by the lecturers (video chat yes/no) and the format of a class (lecture/seminar) were also investigated. In a sample of *N* = 337 students, we tested our hypotheses using structural equation model (SEM). Results confirmed mediation, but not moderation. The use of video chat (video call) seems to facilitate the provision of relatedness support but our data did not show that the format of a class was associated with relatedness. Our findings indicate that both teaching behavior and the technical format used to deliver lectures play important roles in student experiences with online classes. The results are discussed in light of other research conducted during the pandemic.

## Introduction

The COVID-19 pandemic led to the adoption of social distancing measures in many countries and educational institutions were faced with the challenge of teaching their students remotely. In higher education, this resulted in most traditional in-person lessons moving exclusively online, raising questions about how student motivation could best be fostered in that environment. Adding to the difficulties of motivating students in online interactions were the reduced social interactions and increased feelings of social isolation triggered by the pandemic, affecting general well-being negatively (Banerjee and Rai, 2020; Elmer et al., 2020). It is therefore important to investigate the extent to which online classes foster relatedness, a key aspect of teaching quality that has been shown to affect both motivation and vitality (e.g., Standage et al., 2005; Klieme et al., 2009; Taylor and Lonsdale, 2010; Kunter and Trautwein, 2013; Praetorius et al., 2018). Despite the emphasis on the importance of relatedness need support and satisfaction in the educational psychology literature, before the pandemic there were few studies investigating its role in online classes at university level. Examining the extent to which the need for relatedness is supported and satisfied in online education is also valuable beyond the context of the pandemic because it is often argued that despite technical advancements such as video conferencing, online lessons cannot compensate for the complex nature of face-to-face interactions (e.g., Manwaring et al., 2017). This study also looks at the impact of technical facilities (communication channels) and organizational factors (type of class) to better understand students’ experience of relatedness in online classes. Following calls in the discipline to consider individual student characteristics when examining teaching quality (see the opportunity-use model, Helmke, 2017), this study also examines whether differences in the need for relatedness between individuals affect student outcomes in this specific situation.

In the following sections, we first describe the role of relatedness as an important aspect related to students’ intrinsic motivation and their vitality experienced during class. Here vitality is defined as a positive feeling of being alive and energetic, an important aspect of eudaimonic well-being (Ryan and Frederick, 1997; Nix et al., 1999; Niemiec, 2014). Eudaimonic well-being refers to the realization of one’s inherent potential as a human being and is considered a positive subjective state that is the result of striving for self-actualization (Waterman, 2008). We then illustrate why it is important to focus on individual differences in the need for relatedness when trying to understand the effects of relatedness on student outcomes. Finally, we review studies focusing on the role of relatedness and social interaction in online teaching, looking at technical (use of communication channel) and organizational (lecture vs. seminar) preconditions.

## Theoretical Background

### The Role of Student Relatedness

Social interactions with teachers and peers are seen as important prerequisites for learning in education research (Vygotsky, 1978). Self-determination theory is an important framework that systematically addresses the role of how these social interactions affect students (SDT; Deci and Ryan, 1985; Ryan and Deci, 2000, 2017). SDT argues that people benefit from the satisfaction of three innate basic needs, which are presumed to be essential for the optimal functioning of humans and a precondition for health, motivation, and vitality. Those basic psychological needs comprise the need for competence, the need for autonomy, and the need for relatedness. The need for *competence* is defined as the desire for effectiveness and mastery through the interaction with one’s environment, for example, while mastering challenging tasks (Ryan and Deci, 2002). The need for *autonomy* is defined as the need to experience volition, choice, and personal freedom (Vansteenkiste et al., 2010). The third need, the need for *relatedness*, refers to building a sense of community with others that comes with the experience of close and warm relationships characterized by mutual care and concern (Baumeister and Leary, 1995; Deci and Ryan, 2002). The three basic needs are assumed to be innate, and therefore universal regardless of one’s cultural context or gender (Vansteenkiste et al., 2010).

Self-determination theory highlights social environments as facilitators for the satisfaction of the basic needs. In the educational context, teacher behaviors supporting students’ basic needs were often associated with enhanced student motivation, vitality, and performance (e.g., Black and Deci, 2000; Niemiec and Ryan, 2009; Taylor and Lonsdale, 2010; Mouratidis et al., 2013; Stroet et al., 2013; Vergara-Torres et al., 2020). Prior to the pandemic, the examination of the interplay between need support, basic need satisfaction, motivation, and vitality among university students in online learning environments was not, however, a popular subject for investigation (for exceptions see for example Chen and Jang, 2010; Hsu et al., 2019; Wang et al., 2019). In the studies that were conducted, teacher support was usually subsumed in the term “autonomy supportive climate,” often assessed using the learning climate questionnaire (Williams and Deci, 1996) which considers behaviors such as promoting the volition of one’s counterpart by answering questions, and providing choices and options (Williams et al., 1996; Mageau and Vallerand, 2003; Reeve and Jang, 2006; Deci and Ryan, 2008; Vansteenkiste et al., 2010). Besides the emphasis on behaviors fostering autonomy, the construct “autonomy supportive climate” also involves teacher behaviors that support the experience of relatedness in students, such as behaving respectfully toward students. Given the importance of social interaction and the feeling of connectedness for learning (see also Vygotsky, 1978), researchers have become increasingly interested in teacher behaviors that specifically focus on supporting the need for relatedness in education and started exploring it as a separated construct from autonomy support (e.g., Furrer and Skinner, 2003; Standage et al., 2005; Sparks et al., 2015). For example, in a recent study, Sparks et al. (2016) found that physical education teachers’ relatedness supportive behavior affected students’ intrinsic motivation, and this relation was mediated by the satisfaction of students’ need for relatedness. Moreover, the relation between relatedness support and relatedness need satisfaction was shown to be stronger than the relation between an autonomy supportive climate as a more general construct and relatedness satisfaction, justifying the isolated examination of relatedness support.

There are studies investigating the relation between relatedness supportive teacher behaviors, satisfaction of the basic need for relatedness, and student outcomes (Standage et al., 2005; Sparks et al., 2015, 2016), but there is no empirical research on this relationship at university level. This constitutes a significant gap in the literature, as the findings of several studies point to the particular importance of a sense of community and relatedness for the outcomes of university students (e.g., Sheldon and Bettencourt, 2002; Beachboard et al., 2011; Walton and Cohen, 2011; Zainuddin and Perera, 2017; Marksteiner et al., 2019). To summarize, student relatedness satisfaction can be expected to act as a mediator between relatedness support and student motivation and vitality. Although the rationale of SDT would suggest that this relationship is universal and thus equally valid for everyone, research findings indicate that individual differences may play an important role in determining whether someone benefits from the satisfaction of their basic psychological needs (e.g., Schüler et al., 2010). This issue is further elaborated in the next section.

### The Matching Hypothesis – Individual Differences in Relatedness Satisfaction

Recent research indicates that people differ in how much they benefit from the satisfaction of their basic needs. In their work on the matching hypothesis, Schüler et al. (2010), Schüler et al. (2014), Sheldon and Schüler (2011), Schüler and Brandstätter (2013) and Sieber et al. (2016a,b) propose that individual differences in motives (McClelland, 1985) have an impact on how strongly people benefit from basic need satisfaction and basic need support with respect to their motivation and well-being.

To date, researchers have predominantly focused on three motives: the achievement motive (i.e., recurrent concern in surpassing one’s own standards of excellence; McClelland et al., 1953), the affiliation motive (i.e., preference to restore, establish, or maintain close and warm relationships with others, Atkinson et al., 1954; Gable, 2006), and the power motive (i.e., stable concern for influencing and controlling other people; e.g., Winter, 1973; Schultheiss et al., 2005). They further distinguish between implicit and explicit motives, with two independent motivational systems guiding human behavior. Implicit motives “are motivational dispositions […] that operate outside of a person’s conscious awareness” (Schultheiss, 2008, p. 603), Implicit motives develop in the early years and are rarely influenced by social norms and demands (McClelland, 1985; Koestner et al., 1991). They are expressed in long term behaviors and strongly affect non-declarative measures such as task-performance or physiological response (e.g., Schultheiss, 2008; Wegner et al., 2014). By contrast, explicit motives, “self-attributed motives” (McClelland et al., 1989), have a cognitive base and reflect subjective goals, behavioral intentions, and desires that are part of a person’s self-concept (Weinberger and McClelland, 1990). Explicit motives are heavily influenced by the social environment and its expectations. They are associated with controlled behavior and conscious decisions, attitudes, or goals (McClelland et al., 1989; Schultheiss, 2008). Since explicit motives are conscious and reflect an individual’s self-image, they can be assessed using self-reports.

Often, research based on the matching hypothesis is concerned with implicit motives rather than explicit ones. Schüler and colleagues found that the motives function as moderators of need satisfaction effects. For example, people with a high implicit achievement motive benefit more from the satisfaction of the need for competence in terms of flow and well-being, for example, in sports, at the workplace, and in learning environments (Schüler et al., 2010, 2013; Schüler and Brandstätter, 2013). Similar results have been found for people with a high implicit affiliation motive in the sport context: people with a high implicit affiliation motive benefited more from the satisfaction of the basic need for relatedness (Schüler and Brandstätter, 2013).

There is also plenty of evidence supporting the importance of assessing explicit motives when exploring the moderating effects on outcome variables. In an early research project on achievement Harackiewicz et al. (1985) showed that participants’ explicit achievement motive, assessed using self-reporting, moderated the effect between competence need satisfaction and intrinsic interest. More so, Wegner et al. (2014) showed that individuals with a high explicit affiliation motive had a stronger need for affectionate relationships, displayed greater sociability and willingness to cooperate with others, and had more positive attitudes and greater mindfulness toward their teammates than individuals with a less pronounced explicit affiliation motive. A recent study conducted by Schüler and Wolff (2020) further found that participants with a high explicit achievement motive scored lower when a situation did not offer achievement incentives than when a situation did provide such incentives.

These studies show the effectiveness of choosing explicit motives instead of implicit motives in the assessment of motive dispositions and highlight the role of social incentives for the activation of explicit motives. We assumed that the physical isolation during the COVID-19 pandemic means that people are conscious of the social isolation inherent in the situation. Since explicit motives are conscious, we hypothesize that the explicit motives of participants, reflected in respondent behaviors and declarative measures, will influence whether students benefit from the support and satisfaction of their basic need for relatedness. This study did not test the implicit affiliation motive because of the arguments presented above and due to logistical constraints.

### Relatedness in Online Learning Environments

Relatedness and peer interaction play a crucial role in online environments. Thus, it is not surprising that some recent studies highlighted their importance during the COVID-19 pandemic for life satisfaction in general (Teuber et al., 2021), well-being (Yang et al., 2021), motivation (Besser et al., 2020; Camacho et al., 2021), self-regulation (Zhou et al., 2021), and student engagement (Chiu, 2021). However, none of those studies has specifically tested the mediating role of relatedness satisfaction between relatedness support and intrinsic motivation and vitality during online classes. Before the outbreak of COVID-19, most studies on relatedness and peer interaction in online environments focused on a combination of online and on-site teaching (Kramer and Kusurkar, 2017; Manwaring et al., 2017; Wang et al., 2019). In a study of undergraduate and graduate-level online courses, the assignment of a mentor was associated with a greater sense of relatedness during the semester, which in turn had a positive effect on final course grades (Baranik et al., 2017). Research also shows that students’ relatedness experience was significantly lower during online lectures then when taught in person (Butz and Stupnisky, 2016). These results suggest that it is important to examine ways in which technology can facilitate or impede the provision of relatedness.

Research into online communication has examined the role of different types of media used on relational processes in the classroom. Using a variety of media formats not only encourages student–student and teacher–student communication, but also promotes relational processes since the use of media facilitates the transfer and the reception of non-verbal social cues (e.g., facial expressions, gestures, intonation, external features), which are essential for communication. Not using media in the online classroom impedes qualitatively rich communication and increases the time spent clarifying misunderstandings, which are more common when communication lacks non-verbal forms of expression (Short et al., 1976; Baltes et al., 2002). In their social presence theory, Short et al. (1976) define social presence as the degree to which an individual is perceived as “real” in virtual environments, arguing that communication media differ in their degree of social presence due to the varying ability of communication mediums to transmit verbal and visual cues. Conceptualized as the quality of a communication medium, social presence is thought to determine how individuals interact and communicate with one another. The authors posit that people perceive different communication media as having different degrees of social presence, with video conferencing having a higher degree of social presence than, say, audio tools. More importantly, people associate a communication medium with a higher social presence with warmth, closeness, and more personal social connections than a medium lower in social presence, showing that media with a high social presence are more beneficial for relatedness development as they are more personal. Within the educational context, this has led to the assumption that when interaction is restricted, such as by an asynchronous online learning environment, and students do not have the opportunities to create in-depth connections with other course participants, academic performance can be hampered (Moore, 1991; Ahern et al., 1992; Grabinger, 1996; Dowling et al., 2003; Nieuwoudt, 2020; Xi and Gao, 2020).

Other research suggests that the issue is more complex (Roseth et al., 2011). It may be that a speaker adjusts his or her language, intonation, and volume to compensate for the absence of non-verbal social cues (Reicher et al., 1995; Walther, 1996), such as by using emoticons in text-based forms of communication (e.g., email, chats) to transfer affective and interpersonal information (Walther et al., 2005). Within the educational context, Clark (1983) argues that school performance is not directly linked to the richness of the media environment. He proposes that the various types of media merely constitute the medium by which content can be conveyed, but that they do not influence student performance (Clark, 1983; Bernard et al., 2014). This is also in line with theories on the so-called surface and deep structures of learning in online environments, where it is hypothesized that simply looking at surface structures (such as methods used or communication channels) does not provide enough information about processes triggered in the online classroom; that aspects of teaching quality below the surface (i.e., deep structures of learning such as the relatedness support of a lecturer) must also be considered (Voss and Wittwer, 2020).

Summing up, on the basis of media richness theory (Daft and Lengel, 1986) and social presence theory (Short et al., 1976), one could therefore assume that video chat is superior to other communication channels for the provision of relatedness. In line with recent research, one could also suggest that relatedness satisfaction is facilitated by a lecturer using video chat.

Another surface level feature, type of a class (seminar vs. lecture), might also be associated with the provision of relatedness. Based on previous research on the role of different types of a class, it can be assumed that seminars have a positive impact on the relatedness support of lecturers. In seminars, students are more often given the opportunity to contribute to the lesson (Bär et al., 2004; Young et al., 2009), an important aspect of relatedness supportive teaching (Standage et al., 2005). By contrast, lectures are usually more structured and teacher centered. A lecture is also generally directed toward a large number of students and thus naturally more impersonal, allowing for fewer student–student and teacher–student interactions (Garside, 1996; McKeachie, 2002; Black, 2005).

### The Study

This study examines the relation between perceived relatedness support and relatedness satisfaction and student outcomes in the early stage of the COVID-19 pandemic. The expectation is that students who perceive their lecturers as relatedness supportive during online classes experience more relatedness satisfaction and more intrinsic motivation (Hypothesis 1a) and vitality during the lessons in this specific class (Hypothesis 1b). In a second step, the role of individual differences in this relation is examined. Following the tenets of the matching hypothesis, we expect students with a high affiliation motive to benefit more strongly from relatedness satisfaction in terms of motivation (Hypothesis 2a) and vitality (Hypothesis 2b) during online classes.

This study also examines the possible impact of the different teaching conditions adopted by lecturers in response to the pandemic, such as communicating with and without video chat and any differences there may be between lectures and seminars. We hypothesize that the positive relations between perceived relatedness support and relatedness satisfaction are moderated by the inherent interactive potential of the communication channels used (Hypothesis 3). We also expect that the type of a class could influence the degree to which relatedness support is possible, whereas seminars are expected to provide more opportunities for providing relatedness support compared to lectures (Hypothesis 4).

Summing up the present research aims to test the following hypotheses:

H1: Students who perceive their lecturers to be relatedness supportive during online classes experience more relatedness satisfaction which in turn enhances their motivation (H1a) and vitality (H1b).

H2: The affiliation motive moderates the relation between relatedness satisfaction and intrinsic motivation (H2a) and vitality (H2b) (We changed preregistered hypothesis “Students with a high affiliation motive benefit more strongly from relatedness satisfaction in terms of motivation and vitality during online classes” to avoid the causal language).

H3: The use of video chat (video call) moderates the association between perceived relatedness supportive behavior and relatedness need satisfaction (We changed preregistered hypothesis “The use of video chat reinforces the beneficial effect of relatedness supportive behavior on relatedness need satisfaction” to avoid the causal language).

H4: Perceived relatedness support is higher in a seminar compared to a lecture (We changed preregistered hypothesis “Relatedness supportive behavior from a lecturer is facilitated in a seminar compared to a lecture” to avoid the causal language).

**FIGURE 1:**
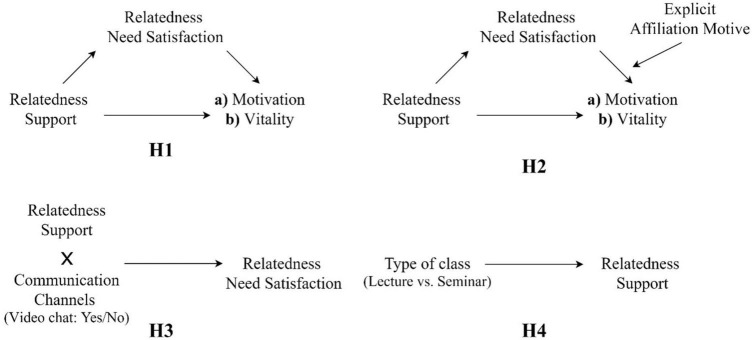
Conceptual models for the tested hypotheses (H1a, H1b, H2a, H2b, H3, and H4). For H1 and H2, motivation and vitality are assessed in separate models.

Figure 1 shows all the hypotheses that are tested in our study.

## Materials and Methods

### Study Design and Sample

A correlational field study with one measurement time point was conducted online. Three weeks after social life in Switzerland was largely restricted and the government mandated that teaching had to move entirely online to prevent the spread of COVID-19, an email was sent to all lecturers at a Swiss university, using the official university contact list, inviting them to participate in the study and forward a questionnaire to their students. The study is part of a large research project, involving both student and lecturer questionnaires (see https://osf.io/jsa35 for more information and the preregistration of the analyses presented below). In total the students’ questionnaire was online for 2 weeks. No reward was offered for participating in the study. Because we could not predict how long COVID-19 measures would continue, we used convenience sampling, which is considered a fast way to recruit participants. The sample size was determined by the number of students who agreed to participate while the study was running. A total of *n* = 538 students agreed to participate in the research; *n* = 103 opted out of the questionnaire before reaching the teaching related questions. To detect participants who rushed through the questionnaire, a relative completion speed index (RSI) was used (see Leiner, 2019). The RSI reflects the sample’s median web-page completion time divided by the individual completion time. Following the recommendations of Leiner (2019), we used a cut-off of 2.0. This means that individuals who were twice as fast as a typical respondent were excluded (*n* = 1). Participants who reported that they did not attend their class or studied at another university were also excluded (*n* = 6). The further reduction of the sample to *n* = 337 is a result of the nested structure of the data. If lecturers taught multiple classes, they were asked to forward the questionnaire to the students of the first class they taught in a regular week. The students were then instructed to answer the questions while thinking about the online lessons of the last 2 weeks regarding that specific class. By class we mean the specific class taught by one single university lecturer. Thus, the students answered the questions with regard to a specific lecture or seminar and not with regard to an entire module or cluster of courses. Every link a specific lecturer sent out had a randomly generated identifying number. This number could be retrieved in the analyses to cluster the students according to which class they attended. To avoid unreliable model estimates, small clusters with *n* < 6 students had to be excluded from the analyses (see, e.g., Bentler and Chou, 1987; Kelley and Maxwell, 2003), resulting in a total of *N* = 337 participants (75% females and 23% males; *M*_*age*_ = 23.96, SD = 7.44, range: 18–73 years) who were enrolled in *n* = 30 classes. Note that for the sake of clarity and in order to avoid causal language, we slightly altered the naming of the preregistered hypotheses in the paper. In contrast to the preregistration, we divide H1 into H1a with motivation as the dependent variable and H1b with vitality as the dependent variable. We deleted the preregistered Hypothesis H2a because we aimed to control for life satisfaction before the outbreak of COVID-19. However, both life-satisfaction before and during the pandemic were each assessed using a single, Likert-type item (i.e., an ordinal scale). Variables measured on an ordinal scale can be problematic to include in parametric statistical models such as structural equation model (SEM) (Jamieson, 2004; Lubke and Muthen, 2004). Additionally, we changed H2b into two new hypotheses: H2a for motivation and H2b for vitality.

### Instruments

*Relatedness support* was measured with five items from Standage et al. (2005), a scale which has been found to have good reliability and validity (Standage et al., 2003, 2005; Sparks et al., 2016). Participants answered how supported they felt in terms of relatedness by their lecturers (e.g., “We experience the lecturer as friendly towards us,” 1 = do not agree at all, 4 = neutral, 7 = fully agree, α = 0.76, ω = 0.87, *M* = 5.95, SD = 0.85).

*Students’ relatedness need satisfaction* during the lesson was measured with six items adapted from Van den Broeck et al. (2010) (e.g., “I feel part of a group,” 1 = do not agree at all, 4 = neutral, 7 = fully agree, α = 0.77, ω = 0.81, *M* = 4.67, SD = 1.25).

*Vitality* was assessed with a German version of the Vitality Scale (Ryan and Frederick, 1997) by Bertrams et al. (2020). This scale has been used often and has been found to be valid and reliable (see for example Sieber et al., 2019; Bertrams et al., 2020). Participants were instructed to respond to the six items describing how they felt during the class (e.g., “I felt energized during the class,” 1 = not at all true, 6 = very true, α = 0.91, ω = 0.93, *M* = 3.82, SD = 1.32).

*Student motivation during the class* is measured with seven items from the Intrinsic Motivation Inventory (IMI, Ryan, 1982), which is a widely used reliable and valid measure (see for example Cortright et al., 2013; Sieber et al., 2016b) (e.g., “I enjoyed the class,” 1 = not at all true, 7 = very true, α = 0.91, ω = 0.93, *M* = 5.09, SD = 1.17).

*The affiliation motive* was measured with six items taken from Schönbrodt and Gerstenberg (2012), which has been previously found to be a valid and reliable measure (e.g., Kaufman et al., 2019), measuring the hope component of the affiliation motive (e.g., “I try to get to know other people,” 1 = strongly disagree, 6 = strongly agree, α = 0.87, ω = 0.90, *M* = 4.34, SD = 0.90).

*The communication channels* were assessed with a single item developed by the authors of the study. University lecturers responded to the question “What methods of communication have you been using since the onset of COVID-19 to interact with your students during the class?” with a multiple response option format: 0 = None, 1 = Provision of material (e.g., PowerPoint slides) on an E-learning platform (e.g., OLAT, Moodle), 2 = Audio recordings, 3 = Video recordings (e.g., podcasts), 4 = Mail, 5 = Text chat and forums (e.g., Microsoft Teams, OLAT, Board.net, Moodle, WhatsApp, Skype), 6 = Audio chat *via* streaming platforms (e.g., Zoom, Microsoft Teams, Skype, Discord), 7 = Video chat *via* streaming platforms (e.g., Zoom, Microsoft Teams, Skype, Discord). In order to investigate Hypothesis 4, we generated a dichotomous variable that only held the information video chat yes vs. no. Of all of students, *n* = 180 attended classes using video chat and *n* = 157 attended classes that were taught using other communication channels (e.g., text chat, provision of materials online, e-mail, etc.). In contrast to the preregistration, we did not analyze different communication channels with respect to their degree of interactivity. Instead, we used a dichotomous variable – video chat/no video chat. This was because most lecturers were using video chat and few were using the other categories. We therefore decided to only contrast video chat to all of the other options.

*The format taught* was assessed with a single item designed by the authors. It captured whether the class was taught as a lecture, a seminar, an exercise group, a tutorial, or a colloquium. As we wanted to compare lectures with seminars, a dichotomous variable was generated. Of all students *n* = 173 attended lectures and *n* = 72 attended a seminar. The remaining 92 students attended other types of classes. This means that the analyses focusing on the role of the type of a class (i.e., Hypothesis 4) are based on a reduced pool of participants (*N* = 245 in 18 clusters).

### Data Analyses

Descriptive analyses were conducted using SPSS Version 25. Results of the confirmatory factor analyses (CFA) for each scale are shown in Table 1.

**TABLE 1:** CFA model fit indices for each scale.

Variable	χ^2^	df	*p*	RMSEA	CFI	TLI	SRMR
Relatedness support	16.0	5	0.007	0.081	0.993	0.986	0.018
Relatedness satisfaction	111.5	9	< 0.001	0.184	0.928	0.880	0.053
Vitality	18.2	5	0.002	0.089	0.998	0.996	0.013
Intrinsic motivation	116.1	14	< 0.001	0.147	0.980	0.970	0.024
Affiliation motive	41.2	9	< 0.001	0.103	0.988	0.980	0.022

We ran a series of SEMs using Mplus software Version 8.4 (Muthén and Muthén, 1998–2017). We used the maximum likelihood estimation with robust standard errors (MLR) and full information maximum likelihood (FIML) to deal with missing data.

Our data have a nested structure with students attending different classes at the university. All of the constructs in the student questionnaire (i.e., relatedness support, relatedness satisfaction, student motivation, vitality, and affiliation motive) were assessed at the individual level. The two surface features, communication channel and class format, were measured at the class level since all students attending the same class experience identical conditions. In Hypotheses 1a, 1b, 2a, and 2b, the unit of interest is the student since they focus on the role of student relatedness and individual differences in relatedness satisfaction. In Hypotheses 3 and 4, which concentrate on surface structures of learning in online environments, the unit of interest is the class level. Therefore, when investigating Hypotheses 1a, 1b, 2a, and 2b, the variables were modeled at the individual level only; the nested structure of the data was taken into account by using the “type = complex” command. For Hypotheses 3 and 4, all variables measured at the student level were modeled simultaneously at the individual and at the class level, but results are reported for the class level only. All the scale indicators in the models are categorical.

The first two models (M1a and M1b) were mediation models. Due to the large number of predictor variables and indicators in our models, we ran the models separately for the two outcomes, intrinsic motivation and vitality. This reduced the number of parameters that had to be estimated, thus avoiding computation issues. All three variables were modeled as latent factors with multiple indicators, taking measurement error into account. The models for Hypothesis 2 (M2a and M2b) were identical to M1a and M1b, except that they also included the affiliation motive as a moderator between relatedness satisfaction and intrinsic motivation. This means that both the latent interaction between the affiliation motive and relatedness satisfaction as well as the factor affiliation motive were included as predictors. The models for Hypotheses 3 and 4 (M3 and M4) were multilevel models, where the student variables were conceptualized at both levels. Modeling these variables as latent factors led to model non-convergence due to the disproportionate relation of the (large) number of parameters to the (small) number of clusters. We therefore calculated the mean across all items belonging to the same scale for each student and included these manifest variables in the models. In M3, relatedness satisfaction was regressed on perceived relatedness support at both levels. At the between level, the moderating role of the communication channel was examined by including this manifest variable as well as the interaction between perceived relatedness support and the communication channel as predictors. In M4, the relation between of the type of a class and perceived relatedness support was investigated by including the type of a class as a manifest variable at the between level. It is important to note that the standardized output is not available in multilevel models with interactions unless the Bayes estimator is used. Therefore, we used unstandardized coefficients for Hypotheses 3 and 4. In section “Results,” we could only report the model fit indices for H4. For the remaining hypotheses, overall model fit calculation was not possible in Mplus (Muthén and Muthén, 1998–2017). The AVE (average variance extracted) for each construct (see Table 2) was satisfactory for all scales except relatedness satisfaction. The convergent validity of the construct is still adequate if the AVE is less than 0.5 but composite reliability is higher than 0.6 (Fornell and Larcker, 1981). Since, the AVE was 0.430 and composite reliability was 0.815 for relatedness satisfaction, we consider convergent validity as satisfactory. Moreover, the square root of AVE for each construct was larger than the correlation coefficients among the constructs, indicating sufficient discriminant validity (Fornell and Larcker, 1981; Tsai et al., 2020).

**TABLE 2:** Means, standard deviations, AVE, square root of AVE, and manifest bivariate correlations of the variables included in the analyses (Pairwise).

Variable	AVE	1	2	3	4	5
1. Relatedness support	0.58	(0.76)				
2. Relatedness satisfaction	0.43	0.20**	(0.66)			
3. Vitality	0.73	0.18**	0.26**	(0.85)		
4. Intrinsic motivation	0.66	0.39**	0.24**	0.47**	(0.81)	
5. Affiliation motive	0.61	0.04	0.18**	0.06	–0.01	(0.78)
*M*		5.95	4.77	3.82	5.09	4.34
SD		0.86	1.24	1.32	1.17	0.90
α		0.76	0.75	0.91	0.91	0.87

**p < 0.01. Note that for some variables the N might be slightly smaller due to missing variables. The numbers in parentheses on the diagonal represent square root of average variance extracted (AVE) of the construct.

## Results

### Descriptive Statistics and Bivariate Correlations

Descriptive statistics and bivariate correlations between the within level variables are presented in Table 2. Correlations between all of the variables in the table were from small to moderate in size. Perceived relatedness support was significantly and positively correlated with relatedness satisfaction, intrinsic motivation, and vitality. However, it was not significantly correlated with affiliation motive. Relatedness satisfaction was significantly and positively correlated with all of the assessed variables. Affiliation motive was only significantly correlated with relatedness satisfaction. The correlations between the outcome variables (i.e., intrinsic motivation and vitality) were significant and positive.

### Main Analyses

#### Hypotheses 1a and 1b

A SEM model was calculated to test whether students’ relatedness satisfaction mediated the relation between perceived relatedness support and their intrinsic motivation (see Figure 1). The results showed that perceived relatedness need support was significantly related to relatedness satisfaction in the expected direction (β = 0.234, SE = 0.066, *p* < 0.001), and that relatedness satisfaction was also significantly associated with intrinsic motivation (β = 0.182, SE = 0.067, *p* = 0.007). The indirect effect of perceived relatedness support on intrinsic motivation was significant (β = 0.043, *p* = 0.025). The total effect was β = 0.454, SE = 0.058, *p* < 0.001, meaning that the indirect effect only made up 9.3% of the total effect. Note that significance testing of indirect effects can be problematic, since indirect effects are not always normally distributed (Hayes, 2018). Applying a bootstrap method can remedy this problem (Eid et al., 2017) but this was not possible for our models, because the large number of parameters resulted in convergence problems.

The mediating role of university students’ relatedness satisfaction between perceived relatedness support and students’ vitality was examined. The results showed that perceived relatedness support was significantly and positively associated with relatedness satisfaction (β = 0.229, SE = 0.067, *p* = 0.001) in the expected direction. Relatedness satisfaction also had a significant positive association with students’ vitality (β = 0.199, SE = 0.079, *p* = 0.011) in the expected direction. The analyses revealed a significant indirect effect of perceived relatedness support on vitality, β = 0.046, *p* = 0.015. The total effect was β = 0.198, SE = 0.066, *p* = 0.003, meaning that the indirect effect made up 23.1% of the total effect.

**FIGURE 2:**
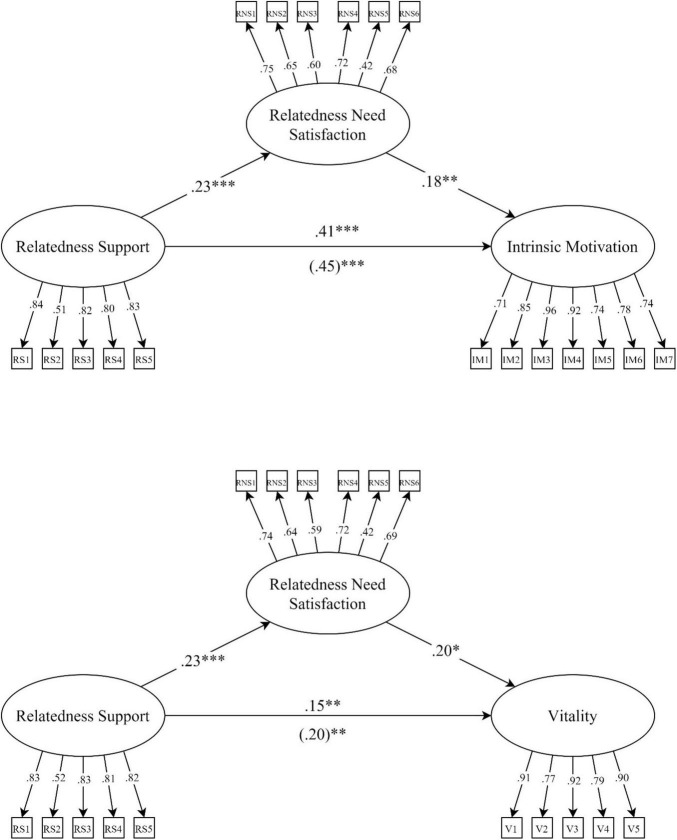
The mediating role of relatedness satisfaction between perceived relatedness support and intrinsic motivation/vitality with standardized coefficients. The numbers in parentheses represent the total effect. *p ≤ 0.05, ^**^p ≤ 0.01, ^***^p ≤ 0.001.

Figure 2 shows the results of the mediation model for Hypotheses 1a and 1b with intrinsic motivation and vitality as outcomes.

#### Hypotheses 2a and 2b

Hypothesis 2 was tested using a moderated mediation analysis. The explicit affiliation motive was expected to moderate the association between perceived relatedness satisfaction and intrinsic motivation (and vitality for H2b, respectively). The analyses revealed that the affiliation motive was not significantly related to intrinsic motivation (β = −0.081, SE = 0.062, *p* = 0.194), and did not statistically significantly moderate the abovementioned relationship (β = −0.025, SE = 0.042, *p* = 0.554).

Similarly, the moderating role of university students’ affiliation motive in relation to their relatedness satisfaction and vitality was examined in a moderated mediation model. Again, the explicit affiliation motive (β = −0.019, SE = 0.058, *p* = 0.737) and the interaction between the affiliation motive and need satisfaction (β = −0.020, SE = 0.047, *p* = 0.669) was not significantly related to vitality.

**FIGURE 3:**
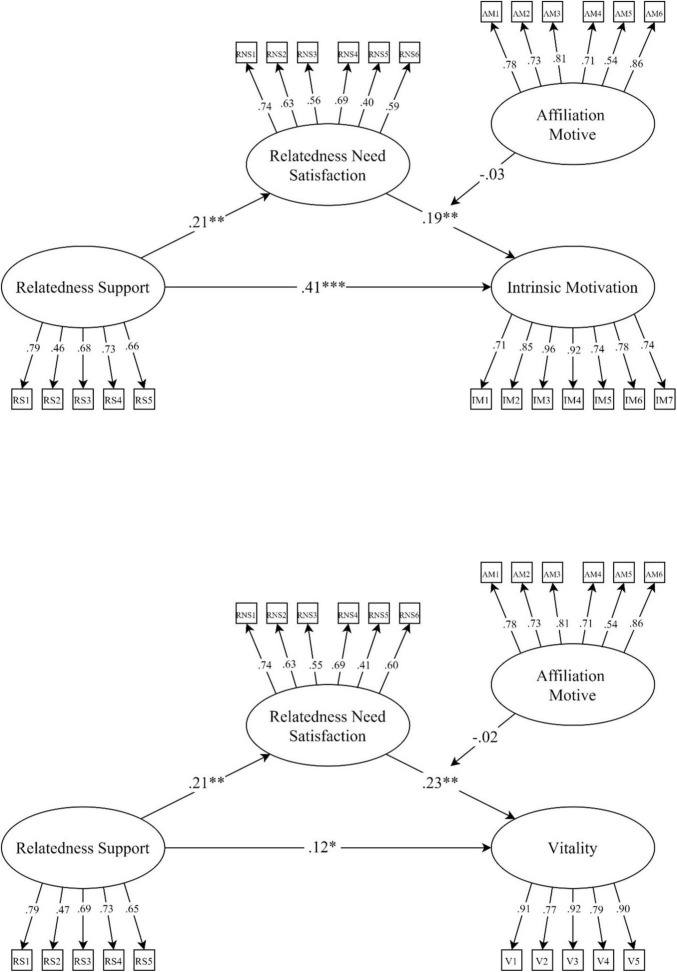
The moderating role of the affiliation motive between relatedness satisfaction and intrinsic motivation and vitality with standardized coefficients. *p ≤ 0.05, ^**^p ≤ 0.01, ^***^p ≤ 0.001.

Figure 3 shows the results of the moderated mediation models for Hypothesis 2 with intrinsic motivation and vitality as outcomes.

#### Hypothesis 3

Hypothesis 3 tested whether the use of video chat facilitated perceived relatedness support. The analyses showed that although the interaction effect lacked statistical significance (*b* = 1.46, SE = 0.773, *p* = 0.060), the inclusion of the interaction between communication channel and relatedness support increased the explained variance of relatedness satisfaction by 18.9%. The unexplained standardized variance of relatedness satisfaction drops from 0.952 in the model without the interaction to 0.763 when interaction is included in the model. This means that the use of video chat seems to facilitate the provision of relatedness support.

#### Hypothesis 4

The relation between type of a class (i.e., seminar vs. lecture) and perceived relatedness support was investigated. The results showed that the class type was not related to perceived relatedness support (*b* = −0.115, SE = 0.175, *p* = 0.512). The hypothesis that seminars enabled more relatedness support than lectures was not supported by the data. The model fit the data well [χ^2^(1) = 0.4, *p* < 0.49; CFI = 1, RMSEA = 0, SRMR_*within*_ = 0, SRMR_*between*_ = 0.003].

## Discussion

This study demonstrates that relatedness support provided by lecturers is associated with students’ relatedness satisfaction, which in turn is associated with enhanced motivation and vitality in online classes. This relationship was found during times of social isolation when social distancing measures were enforced to prevent the spreading of COVID-19. Feelings of isolation and reduced interaction were among the most significant effects resulting from universities switching to online teaching and learning (Banerjee and Rai, 2020; Elmer et al., 2020). A closer look at our results reveals that the provision of relatedness is especially fruitful when provided *via* video. Interestingly, the extent to which lecturers were perceived to support students’ relatedness did not differ between lectures and seminars. Moreover, contrary to our expectations, the affiliation motive was not a statistically significant moderator between need satisfaction and motivation (and vitality, respectively).

The results relating to Hypotheses 1a and 1b confirm that students’ perceived relatedness support from their lecturers relates positively to their experience of intrinsic motivation and vitality during classes, and that relatedness need satisfaction mediates this relationship. Our results further substantiate existing research which suggests that basic psychological needs explain motivation and vitality within the school context (e.g., Black and Deci, 2000; Niemiec and Ryan, 2009; Mouratidis et al., 2013; Stroet et al., 2013; Vergara-Torres et al., 2020). While studies which were conducted within the theoretical framework of SDT during the COVID-19 pandemic have examined relatedness along with competence and autonomy as a mediator between an autonomy-supportive climate and student outcomes (see Shah et al., 2021), the relationship between specifically relatedness supportive behaviors and relatedness satisfaction has largely been neglected in this context so far. This is insofar interesting, as studies conducted in Israel (Besser et al., 2020) and China (Yang et al., 2021; Zhou et al., 2021) have shown that the satisfaction of the need for relatedness and related constructs (e.g., sense of belonging) was especially important in this period for university students. Moreover, in an intervention study relatedness support specifically fostered eighth and ninth graders’ relatedness satisfaction during the pandemic (Chiu, 2021). This is also in line with studies conducted before the pandemic (e.g., Sparks et al., 2016, 2017). By linking relatedness support and relatedness satisfaction, this study theoretically contributes to understanding the interplay of these factors in the university context in online classes. Most importantly, however, our study shows that even in a setting with obstacles to relatedness, teacher behaviors fostering this specific need are important. By adopting a mediation perspective, this research shows the importance of focusing not only on teacher behaviors, but also on how students respond to what teachers do in terms of relatedness satisfaction. This is consistent with an opportunity and use notion discussed in current theoretical models (see the opportunity-use model, Helmke, 2017, and the MAIN-TEACH model, Charalambous and Praetorius, 2020). Within such models it is postulated that it is central to examine not only the learning opportunities provided within a learning environment (i.e., the support of relatedness), but also the use of these opportunities by the students (i.e., relatedness satisfaction) in order to understand what defines high-quality teaching. Following this reasoning, it can be assumed that relatedness support is only effective if it is able to satisfy the students’ need for relatedness.

Interestingly, however, there are also studies that do not confirm the positive effect of relatedness satisfaction on student outcomes. In their studies conducted at different school levels and across different cultural contexts, Holzer et al. (2021a,b, c) found mixed results regarding the relation between relatedness and intrinsic motivation. For one, the relation between relatedness and intrinsic motivation differed across cultures. For example, a positive relation between relatedness and intrinsic motivation was found for university students in Finland but not in Austria (Holzer et al., 2021c). Further, it is important to point out that they defined relatedness as a more general construct, which could explain why their results were discrepant from our study. Our findings thus highlight the importance of the specific context in which need satisfaction is assessed when looking at the relationship with student outcomes. By conducting a study in Switzerland, the present research adds this cultural perspective to the literature. For future research it will be interesting to further examine these relations in different settings and countries.

The second hypothesis extends the above findings by considering the affiliation motive as an important moderator of the mediation model described above (Hypothesis 2). However, contrary to our hypotheses, the interaction between relatedness satisfaction and the explicit affiliation motive did not have a statistically significant relation to intrinsic motivation and vitality during online classes (Hypothesis 2). These findings do not fit with the idea of a matching hypothesis (see Schüler et al., 2010). The lack of a statistically significant effect in our study could be due to three reasons: first, there is reason to assume that the emergence of the pandemic as an unfamiliar and life changing event has influenced participants’ personal perception and response behavior. Although it is generally believed that individual differences in personality become more apparent during periods of social and environmental change (Caspi and Moffitt, 1993), other studies show the opposite (e.g., Stewart, 1982; Stewart and Healy, 1985). Stewart (1982) found that the effects of individual differences are minimized when participants enter new and unfamiliar periods of life. Indeed, it has been argued that “external life changes [are] a major catalyst for personality change” (Stewart and Healy, 1985, p. 140), resulting in distorted results when assessing personality variables during those periods. From this perspective it can be argued that the novelty of the situation had not yet affected individuals’ differences in affiliation motive, reducing the differences between participants. To investigate this further, it would be interesting to continue to study the affiliation motive in online classes, since it can be assumed that people have become accustomed to the transition by now. To find out whether new situations affect the impact of explicit motives in university classes, one could focus on transitions, such as the transition from high school to university, which is associated with difficulties and challenges (Anderson et al., 2000).

Second, cultural disasters, crises, and epidemics have been shown to evoke people’s helpfulness (James, 1911; Fritz, 1961; Anderson, 2020; Bregman, 2020). Global crises such as the COVID-19 pandemic act as incentives to do good as they “require us to act, and act altruistically, bravely, and with initiative in order to survive or save our neighbors, no matter their wealth or how they vote” (Solnit, 2009, p. 7; see also Bregman, 2020; ONS, 2020; Populus, 2020). This social engagement and spirit of goodwill might have caused lecturers and students to be more aware of the need to support one another and share acts of kindness in order to maintain a sense of community and togetherness at a time of social isolation, independent of their individual affiliation motive.

Third, explicit motives, assessed using self-reports, are conscious reflections of what a person desires, and thus can be susceptible to social expectations and demands as well as inaccurate self-theories (McClelland et al., 1989; Schultheiss, 2008). Several researchers found that they were important predictors of behavior (e.g., Wegner et al., 2014; Schüler and Wolff, 2020). However, some researchers question their suitability as moderators between need satisfaction and motivational outcomes. Ryan and Deci (2000) claim that “part of the problem with assessing need strength as a moderator of the effects of satisfying the need also results from confusion between needs and their conscious representations” (Ryan and Deci, 2000, p. 328), criticizing studies using self-reported motives as moderators (e.g., Harackiewicz et al., 1985; Richer et al., 2002). Compared to their explicit counterparts based on cognition, implicit motives are based on affect and can thus better represent deep-rooted desires and preferences (cf. section “The Matching Hypothesis – Individual Differences in Relatedness Satisfaction”). Variables such as motivation and vitality lead to spontaneous, affect based decisions that are rarely influenced by social pressure or expectations and demands established by the environment (McClelland et al., 1989). By contrast, responding to items assessing the explicit affiliation motive requires greater cognitive effort as they address not only the individual but also others, activating social values and pressures. This can cause social desirability effects, distorting participants’ perceptions and ultimately their responses. The lack of alignment between intrinsic measures of motivation and vitality and the explicit affiliation motive is therefore considered to be a viable reason for the absence of this effect in this study. Future research could therefore assess implicit and explicit motives in parallel to investigate specific effects on different outcomes. Explicit motives could be assumed to have a stronger influence on aspects such as attitudes toward online instruction (see for example Brewer and Klein, 2000), and implicit motives could be assumed to have a stronger influence on intrinsic motivation and vitality (see for example Sieber et al., 2016b). Further, by recording implicit and explicit motives in parallel, effects of congruence between those motivational systems on satisfaction and motivation in online classes could be explored. Positive effects on well-being and motivation can be expected when these motivational systems are in congruence (see for example Baumann et al., 2005; Schüler et al., 2008).

Although individual differences in the affiliation motive were not found to play an important role in this study, the communication channel chosen to deliver the lesson did affect how successful lecturers were in supporting their students’ relatedness. Although not statistically significant, the inclusion of the interaction between communication channel and relatedness support increased the explained variance of relatedness satisfaction by 18.9%, which speaks in favor of our Hypothesis 4. The change in explained variance could be interpreted as a sign that the interaction between the communication channel and relatedness support should be considered when predicting relatedness satisfaction. This result, however, remains inconclusive, and needs further studies with more power, that is, larger sample sizes. Indeed, choosing video chat seemed to enhance the effects of a relatedness supportive teaching style. Our finding contributes to the recent studies showing that hosting interactive real time lessons with video chat enhances positive experiences and motivation in online classes (Krammer et al., 2020; Fabriz et al., 2021) and student relatedness satisfaction (Chiu, 2021). Our finding also accords with media richness theory (Daft and Lengel, 1986) and social presence theory (Short et al., 1976) which posit that the use of media high in social presence in the classroom not only improves communication but also promotes relationship processes as the use of such media facilitates the transmission and reception of non-verbal social cues that are important in the personal exchange (e.g., facial expressions). A very important aspect of the present study is that the interaction of communication channel and lecturer behavior was investigated in a moderation hypothesis. This approach takes into account a highly topical theoretical discussion on surface and deep structures, which states that the isolated view of either surface or deep structures might not be sufficient to explain the effects of teaching on students. Our finding suggest focusing on the interplay of those aspects, an issue that has recently been addressed by several researchers in the field (Decristan et al., 2020; Hess and Lipowsky, 2020).

Finally, our analyses did not confirm that seminars lead to more relatedness supportive lecturer behavior (Hypothesis 4). One reason for this result can be that the class type does not convey much information about what actually happens in a lesson. Like communication channels, class type can be considered a surface aspect of teaching, but the effects probably largely depend on which teaching methods lecturers use. What constitutes a seminar or a lecture can also differ across disciplines as well as between lecturers. While lectures are assumed to be less learner-centered, giving students fewer opportunities for direct involvement (see Bär et al., 2004), some lectures might allow for student interaction and group assignments (Murphy and Sharma, 2010). Although controlling for these influences would be of great interest, such analyses in this study were not possible due to the small sample size at the between level.

### Practical Implications

Our results suggest that even in environments that are suboptimal with respect to relatedness, enhancing relatedness supportive behavior by lecturers is desirable. This could be achieved by encouraging the use of relatedness supportive techniques (Sparks et al., 2016; Gruno et al., 2018), fostering cooperation and teamwork between students (Sparks et al., 2016), creating a climate of mutual acceptance, respect, caring, and support (Standage et al., 2005), hosting interactive real-time lessons where students can contribute to the lesson, or creating small teacher–student support groups (see Chiu, 2021). Our results also indicate that students can be motivated and feel vital when their relatedness satisfaction is supported. Lecturers should be open to discuss how best to satisfy their students’ relatedness need during online lessons. Our results on the use of video chat show that the targeted use of appropriate tools can enhance the beneficial effects of relatedness support on relatedness satisfaction. To this end, modern teaching techniques specifically designed for online lessons could be employed, such as discussions in breakout rooms, creating diagrams and mind maps on online whiteboards, conducting online polls, surveys, and quizzes, writing a class blog to encourage content related exchange, and maintaining discussion boards for individual lesson topics (see Krammer et al., 2020; Chiu, 2021; Wut and Xu, 2021).

### Limitations

Our study used a convenience sample. Moreover, our study is cross-sectional and correlational in nature as the measurements were made after the changes due to COVID-19 were implemented. Future research on higher education should investigate the hypothesized relations longitudinally. Further studies would ideally compare on-site and online teaching in university settings in experimental designs or interventions (for a recent study examining different digital support strategies for need satisfaction during COVID-19 with eighth and ninth graders, see Chiu, 2021). Experimental designs in applied domains could actively manipulate lecturers’ relatedness support by, for example, instructing lecturers to behave in a relatedness-supportive way, encouraging a respectful interaction based on mutual interest and cooperation (see Standage et al., 2005; Sparks et al., 2016; Gruno et al., 2018).

Another limitation of the study is the small cluster size (*n* = 30). Although, we took the nested structure of the data into account, future research should conduct multilevel SEM using a larger cluster size. The study also used self-reporting instruments for all assessed variables. For future research we recommend including other types of measurement such as observer- or teacher-ratings (for further discussion on that topic see Fauth et al., 2020). Our study focused on the beneficial effects of relatedness support and the subsequent satisfaction of the basic need for relatedness. Yet, some authors emphasize the importance of distinguishing between satisfaction and the thwarting of a basic need (Bartholomew et al., 2011; Costa et al., 2015; Shah et al., 2021). Their research on differentiating between the satisfaction and thwarting of a basic need highlights that the satisfaction of a basic need predicts positive outcomes, while the thwarting of a basic need predicts negative aspects more effectively. In the situation elicited by COVID-19, in which relatedness might be restricted due to the circumstances discussed in our paper, the assessment of need thwarting would be of great interest for future research, especially when predicting negative outcomes, such as burnout or depression (see Bartholomew et al., 2011).

Although our results showed no statistically significant effect of the affiliation motive on relatedness satisfaction, future research should use other measures to assess the affiliation motive. For example, following the reasoning of Schüler and Brandstätter (2013) and Schüler et al. (2017), implicit motives rather than explicit motives might affect how strongly someone benefits from the satisfaction of the basic need for relatedness. Individuals with a high implicit affiliation motive benefit more strongly from the support and the experience of relatedness (Schüler and Brandstätter, 2013; Schüler et al., 2017). In contrast, when confronted with affiliation-related goal instructions, individuals with a low affiliation motive are less motivated, report lower levels of well-being, and show poorer performance (Schüler et al., 2017). Investigating whether relatedness support has equally positive effects for all individuals in online classes is thus of great importance, especially given that there is room for variation in terms of the surface structure.

## Conclusion

Our study provides evidence that supporting relatedness is beneficial for motivation and vitality during online classes in a university setting when distance learning is mandated and that this relation is mediated by the satisfaction of students’ need for relatedness. Moreover, the communication channels used seem to play an important role in how successful lecturers are in supporting students’ relatedness, indicating that it is important to look at the interaction of surface and deep structures of instruction when aiming at understanding student outcomes. Therefore, this research supports recent calls to consider the interplay between aspects of surface and deep structures.

## Data Availability Statement

The raw data supporting the conclusions of this article will be made available by the authors, without undue reservation.

## Ethics Statement

Ethical review and approval was not required for the study on human participants in accordance with the local legislation and institutional requirements. The patients/participants provided their written informed consent to participate in this study.

## Author Contributions

VC-S conceived and designed the study and collected the data with JH. A-KP provided advice on the study design and questionnaire. CK carried out main data analysis and conducted additional analyses for the second and third versions of the manuscript. JH and AAC created the descriptive analyses, tables, and figures. VC-S drafted the first version of the manuscript. AAC and VC-S drafted the second and third versions of the manuscript. All authors were involved in writing the manuscript.

## Conflict of Interest

The authors declare that the research was conducted in the absence of any commercial or financial relationships that could be construed as a potential conflict of interest. The handling editor declared a past collaboration with one of the authors A-KP.

## Publisher’s Note

All claims expressed in this article are solely those of the authors and do not necessarily represent those of their affiliated organizations, or those of the publisher, the editors and the reviewers. Any product that may be evaluated in this article, or claim that may be made by its manufacturer, is not guaranteed or endorsed by the publisher.
